# An integrated method for phenotypic analysis of wheat based on multi-view image sequences: from seedling to grain filling stages

**DOI:** 10.3389/fpls.2024.1459968

**Published:** 2024-08-19

**Authors:** Shengxuan Sun, Yeping Zhu, Shengping Liu, Yongkuai Chen, Yihan Zhang, Shijuan Li

**Affiliations:** ^1^ Agricultural Information Institute, Chinese Academy of Agricultural Sciences, Beijing, China; ^2^ Institute of Digital Agriculture, Fujian Academy of Agricultural Sciences, Fuzhou, Fujian, China; ^3^ College of Letters & Science, University of California, Davis, Davis, CA, United States

**Keywords:** wheat plant, multi-view stereo reconstruction, phenotype analysis, point cloud processing, growth dynamics analysis

## Abstract

Wheat exhibits complex characteristics during its growth, such as extensive tillering, slender and soft leaves, and severe organ cross-obscuration, posing a considerable challenge in full-cycle phenotypic monitoring. To address this, this study presents a synthesized method based on SFM-MVS (Structure-from-Motion, Multi-View Stereo) processing for handling and segmenting wheat point clouds, covering the entire growth cycle from seedling to grain filling stages. First, a multi-view image acquisition platform was constructed to capture image sequences of wheat plants, and dense point clouds were generated using SFM-MVS technology. High-quality dense point clouds were produced by implementing improved Euclidean clustering combined with centroids, color filtering, and statistical filtering methods. Subsequently, the segmentation of wheat plant stems and leaves was performed using the region growth segmentation algorithm. Although segmentation performance was suboptimal during the tillering, jointing, and booting stages due to the glut leaves and severe overlap, there was a salient improvement in wheat leaf segmentation efficiency over the entire growth cycle. Finally, phenotypic parameters were analyzed across different growth stages, comparing automated measurements of plant height, leaf length, and leaf width with actual measurements. The results demonstrated coefficients of determination ( $R^{2}$ ) of 0.9979, 0.9977, and 0.995; root mean square errors (RMSE) of 1.0773 cm, 0.2612 cm, and 0.0335 cm; and relative root mean square errors (RRMSE) of 2.1858%, 1.7483%, and 2.8462%, respectively. These results validate the reliability and accuracy of our proposed workflow in processing wheat point clouds and automatically extracting plant height, leaf length, and leaf width, indicating that our 3D reconstructed wheat model achieves high precision and can quickly, accurately, and non-destructively extract phenotypic parameters. Additionally, plant height, convex hull volume, plant surface area, and Crown area were extracted, providing a detailed analysis of dynamic changes in wheat throughout its growth cycle. ANOVA was conducted across different cultivars, accurately revealing significant differences at various growth stages. This study proposes a convenient, rapid, and quantitative analysis method, offering crucial technical support for wheat plant phenotypic analysis and growth dynamics monitoring, applicable for precise full-cycle phenotypic monitoring of wheat.

## 1 Introduction

With the rapid growth of the global population and the intensification of environmental challenges, global agriculture faces serious challenges of ensuring food safety, necessitating the breeding of high-yield, stable crop genotypes that can adapt to climate change to secure future food and fuel supplies (Furbank and Tester, 2011). As one of the three major global food crops, wheat plays a crucial role in global food safety (Erenstein et al., 2022). Accurate acquisition of wheat phenotypic traits is of great practical importance for crop growth assessment, yield estimation, strain breeding and variety improvement (Fiorani and Schurr, 2013; Araus and Cairns, 2014). However, traditional manual measurements are inefficient and destructive in modern plant sciences, making it difficult to capture small changes in crop growth, thereby impacting breeding efficiency and progress (Das Choudhury et al., 2019; Liu et al., 2023). Therefore, there is an urgent call to develop advanced phenotypic data acquisition techniques (Yang et al., 2020; Li et al., 2022) to obtain plant phenotypic parameters in a non-destructive, rapid, efficient, and precise manner (Xie and Yang, 2020).

To this end, researchers have widely applied various high-tech sensors to collect plant morphological phenotypic information, including RGB cameras (Wu et al., 2022b; Wei et al., 2024; Yang et al., 2024), depth cameras (Teng et al., 2021; Ma et al., 2022; Liu et al., 2023), LiDAR (Sun et al., 2018; Forero et al., 2022; Liao et al., 2022), and three-dimensional (3D) scanners (Chenxi et al., 2022; Wu et al., 2022a), etc. Depth cameras offer high precision and real-time capabilities; however, they are constrained by range, environmental factors, typically lower resolution, complex data processing, and higher costs. Similarly, LiDAR and 3D scanners provide high precision and accuracy in capturing detailed 3D point clouds, but they are limited by the need for high computational resources, elevated costs, and sensitivity to environmental conditions (Rongsheng et al., 2021; Yu et al., 2024). In contrast, RGB cameras are widely used due to the advantages of low cost, ease of use, reliable imaging, and strong environmental adaptability (Paproki et al., 2012; Wu et al., 2022b). However, 2D image data from RGB cameras often face challenges in addressing the complicated shielding between plant organs, and the accuracy of morphological parameter extraction is frequently limited by shooting angles. To overcome these limitations, researchers have developed the image sequence-based SFM-MVS (Structure-from-Motion, Multi-View Stereo) reconstruction technique, which generates a point cloud through multi-view image reconstruction, capturing rich and abundant 3D information of the plant and considerably improving measurement accuracy (Yang et al., 2024). This technique has been successfully applied to crop phenotyping, enabling the construction of high-precision 3D models and accurate extraction of phenotypic parameters for various crops such as corn (Wu et al., 2020; Li et al., 2022), cotton (Hao et al., 2024), soybean (He et al., 2023), sugar beet (Xiao et al., 2020, 2021), and more.

Compared to other plants, current 3D reconstruction techniques for wheat and rice are mainly applicable to early-stage plants with relatively simple structures or few tillers. The generalization and robustness of these techniques in dealing with the entire growth cycle of wheat still require improvement (Gu et al., 2024). In recent years, substantial progress has been achieved and accomplished in wheat phenotyping research. For example, Fu et al. (Fu et al., 2021) used Mask R-CNN technology to extract leaf length and plant height from 2D images of wheat seedlings, with coefficients of determination ( $R^{2}$ ) of 0.87 and 0.98, respectively. Zheng et al. (Chenxi et al., 2022) manually labeled key points using a 3D digitizer to obtain 3D data of wheat and gleaned phenotypic parameters during standing, jointing, and heading stages, with $R^{2}$ for plant height, leaf length, and leaf width being 0.98, 0.87, and 0.75, respectively.

Additionally, studies based on SFM algorithms for multi-view image reconstruction have gained advanced developments. For example, Duan et al. (Duan et al., 2016) successfully reconstructed a 3D model of wheat at the seedling stage using the SFM algorithm, achieving an $R^{2}$ of 0.98 for leaf length. The MVS-Pheno V2 platform, developed by Wu et al (Wu et al., 2022b), utilized the SFM algorithm to focus on analyzing the shoots of low plants and acquired wheat point clouds from the beginning of returning to green stage to booting stage with notable success, obtaining $R^{2}$ for plant height, leaf length, and leaf width reaching 0.9991, 0.9949, and 0.9693, respectively. Combining with multi-view images and new virtual design method, Gu et al. (Gu et al., 2024) reconstructed a wheat plant model at heading stage, and attained plant height, crown width, plant leaf area, and coverage parameters, with an average $R^{2}$ of 0.78. Wheat exhibits complicated changes throughout its growth cycle (Liu et al., 2023), such as dense tillers, thin and soft leaves, and cross-obscuration between organs, making phenotypic monitoring throughout its full cycle a challenging and arduous task. Although current research has yielded some results, existing methods still require advancement to continuously track wheat growth dynamics and accurately extract phenotypic parameters.

In view of this, this study designed a comprehensive SFM-MVS-based methodological workflow to process and segment wheat point clouds and automatically extract phenotypic parameters, covering the entire growth period (from seedling to grain-filling stages) for three varieties. First, we propose a new method for point cloud preprocessing that improves algorithm efficiency by using centroid-based segmentation to enhance the Euclidean clustering algorithm. Meanwhile, in the preprocessing workflow, noise is successfully removed through scale reduction, coordinate system correction, and combining color and statistical filtering algorithms, resulting in high-quality, 1:1 restored wheat point cloud models.

To enhance the efficiency of stem and leaf segmentation, a region growing algorithm was employed to automatically segment wheat stems and leaves, achieving notable success and improved segmentation efficiency to some extent. Important phenotypic parameters such as wheat plant height, leaf length, and leaf width were also extracted and compared with manual measurements for an in-depth assessment of the model reconstruction’s accuracy. Ultimately, this study analyzed changes in growth dynamics throughout the growth cycle by extracting phenotypic parameters such as plant height, convex volume, plant surface area, and crown area, and ANOVA was performed according to different varieties in order to accurately demonstrate and differentiate the significant differences among different varieties of wheat at each growth stage.

## 2 Materials and methods

**Figure 1:**
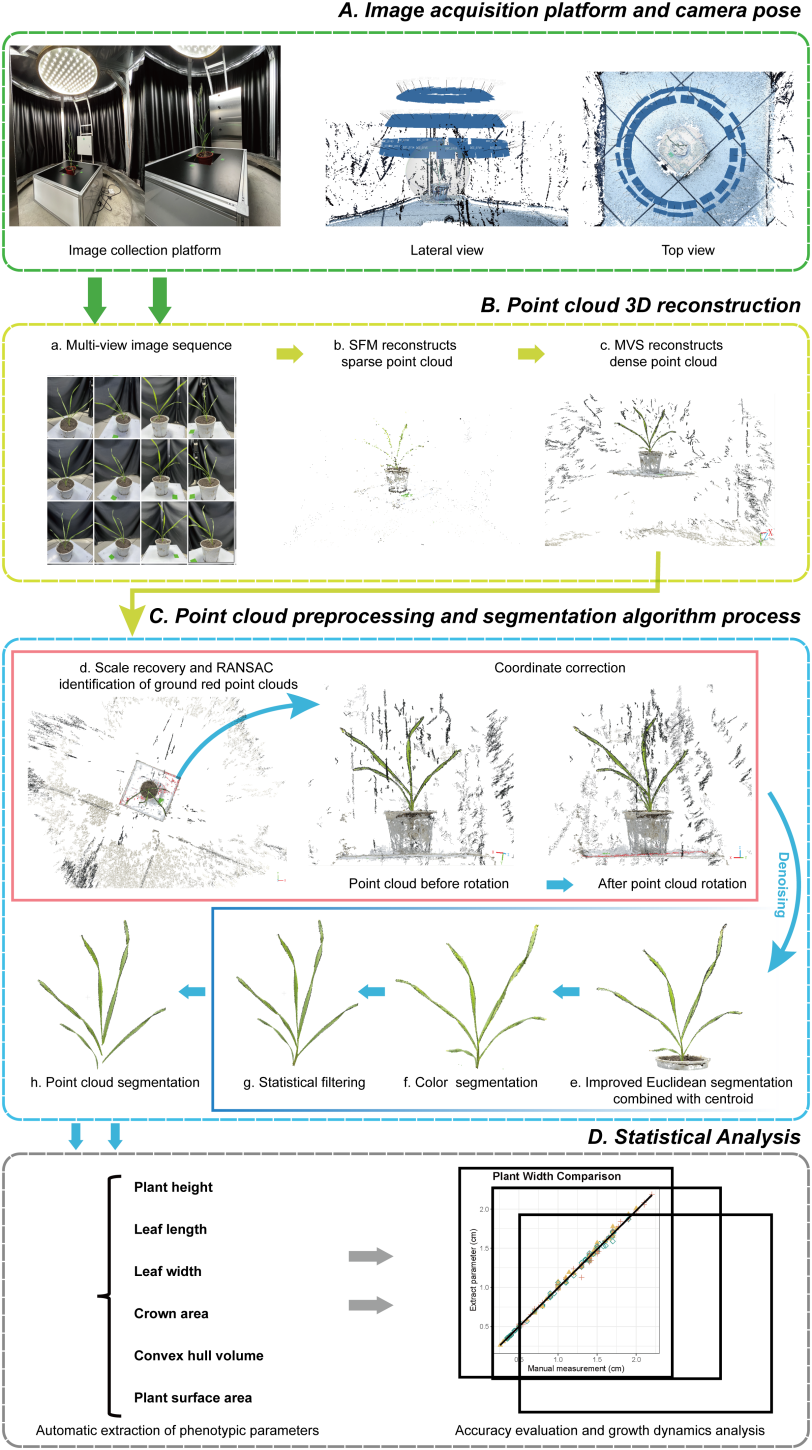
Overall process of phenotypic parameter extraction and analysis of wheat. **(A)** Image acquisition platform and camera pose. **(B)** Point cloud 3D reconstruction using SFM-MVS (a-c). **(C)** Preprocessing of point cloud data, including scale recovery, coordinate correction (d), noise removal (e-g), and segmentation (h). **(D)** Statistical analysis.

In this study, the 3D morphology of wheat plants with multiple life cycles was reconstructed using the SFM-MVS (Structure-from-Motion, Multi-View Stereo) algorithm, selecting three wheat cultivars with different plant characteristics as subjects. Main steps are introduced as follows (**Figure 1**):(1)Sample field-grown wheat plants and transplant them into pots without damaging their three-dimensional morphological structure; (2) Acquire multi-view image sequences of the plants using the image acquisition platform; (3) Reconstruct the three-dimensional point cloud using the SFM-MVS algorithm; (4) Restore the real-world scale of the point cloud by measuring the width of the marker stickers and rotate the point cloud to the real-world coordinate system using the RANSAC algorithm; (5) Use improved Euclidean clustering, color filtering, and statistical filtering to remove noise; (6) Perform stem-leaf segmentation using the region growing algorithm; (7) Down-sample the leaf point cloud and fit the leaf midrib with a local polynomial function; (8) Extract plant height, leaf length, and leaf width automatically to compare with the measured data to assess the accuracy of the wheat model; (9) Obtain crown width, convex volume, and plant surface area, and combine with ANOVA based on species effect to reveal differences and growth dynamics among different varieties and periods. Detailed descriptions of the specific experiments and the technologies and methods used are provided in the following sections.

### 2.1 Experimental environment

Three winter wheat cultivars with distinct plant characteristics were used in this study: FuMai1 (FM), PinHong1 (PH), and PinBai1 (PB). FM, a common commercial variety, has a compact plant type, moderate tillers, and average uniformity. PH and PB, intermediate varieties bred by the Fujian Academy of Agricultural Sciences, differ in their plant types; PH has a loose plant type with more tillers, while PB has a moderately compact plant type with fewer tillers. The experiment was conducted at the National Digital Plantation Innovation Center test field of the Institute of Agricultural Information, Chinese Academy of Agricultural Sciences (26°07′N, 119°20′E). Wheat was sown on November 27, 2023, at a density of 10 kg per mu. Each variety was planted in two plots, totaling six plots, with each plot divided into three rows spaced 30 cm apart. Adequate water and fertilizer were provided throughout the growth period. Plant samples were collected at the seedling, tillering, jointing, booting, heading, and grain filling stages, with four replicates per variety. The wheat samples were quickly transplanted into pots, and adequate water was added to prevent leaf wilting. Image sequences were collected after the wheat morphology stabilized.

An automated multi-view image acquisition platform, consisting of a rotating imaging arm, a lifting platform, supplementary lights, and a black background cloth, was self-constructed for the entire growth period of wheat (**Figure 1A**). Three RGB industrial cameras, each with 20 million effective pixels, were mounted on a rotating imaging arm, spaced 25 cm apart. The height of the plants was adjusted using a lifting platform to accommodate the cameras. The rotating arm with cameras rotated around the plants, capturing image sequences at intervals of 7° to 10° over a 360° range, with the camera and plant positions referenced, as shown in **Figure 1A**. After imaging, the plant height of the sampled wheat was measured immediately, and 2-3 leaves were randomly selected for measuring leaf length and width, with the leaves marked for subsequent verification.

### 2.2 SFM-MVS based 3D reconstruction and point cloud preprocessing

In this paper, Agisoft Metashape (version 2.1.0, Agisoft LLC, St. Petersburg, Russia), a software integrating SFM (structure from motion) and MVS (multi-view stereo) algorithms, was used for 3D point cloud reconstruction of wheat image sequences. Image-based 3D reconstruction converts a set of 2D images into a 3D model (Aharchi and Ait Kbir, 2020), utilizing SFM and MVS techniques, and is considered a powerful method (Kholil et al., 2021) capable of generating high-quality 3D models from 2D images.

SFM-MVS works as follows: first, the image sequence is analyzed by SFM to obtain the camera pose and create a sparse 3D point cloud. This step involves feature detection, matching, and bundle adjustment to estimate the camera pose and the sparse 3D point cloud (Westoby et al., 2012). Subsequently, MVS generates a dense point cloud by estimating depth information for each pixel, utilizing multiple viewpoints to improve accuracy (Furukawa et al., 2015). This process includes techniques such as stereo matching for calculating pixel differences and generating depth maps, which are then fused to create a coherent representation of the scene geometry, resulting in high-quality 3D reconstruction.

During the point cloud reconstruction process, the generated point cloud usually differs in scale and coordinate system from the real world. To accurately restore the wheat point clouds to a 1:1 scale, two key steps were taken. First, the scaling factor was obtained by repeatedly measuring the width of marker stickers in the point cloud and comparing it to the real sticker width, and then adjusting the scale of the point to match the real-world dimensions. Second, the ground is detected using the RANSAC (Random Sample Consensus) algorithm (Fischler and Bolles, 1981), and the rotation matrix of the point cloud is calculated based on the detection results. By rotating the point cloud to the real-world coordinate system, it was ensured that the plants were aligned along the Z-axis from top to bottom (**Figure 1C.d**).

**Figure 2:**
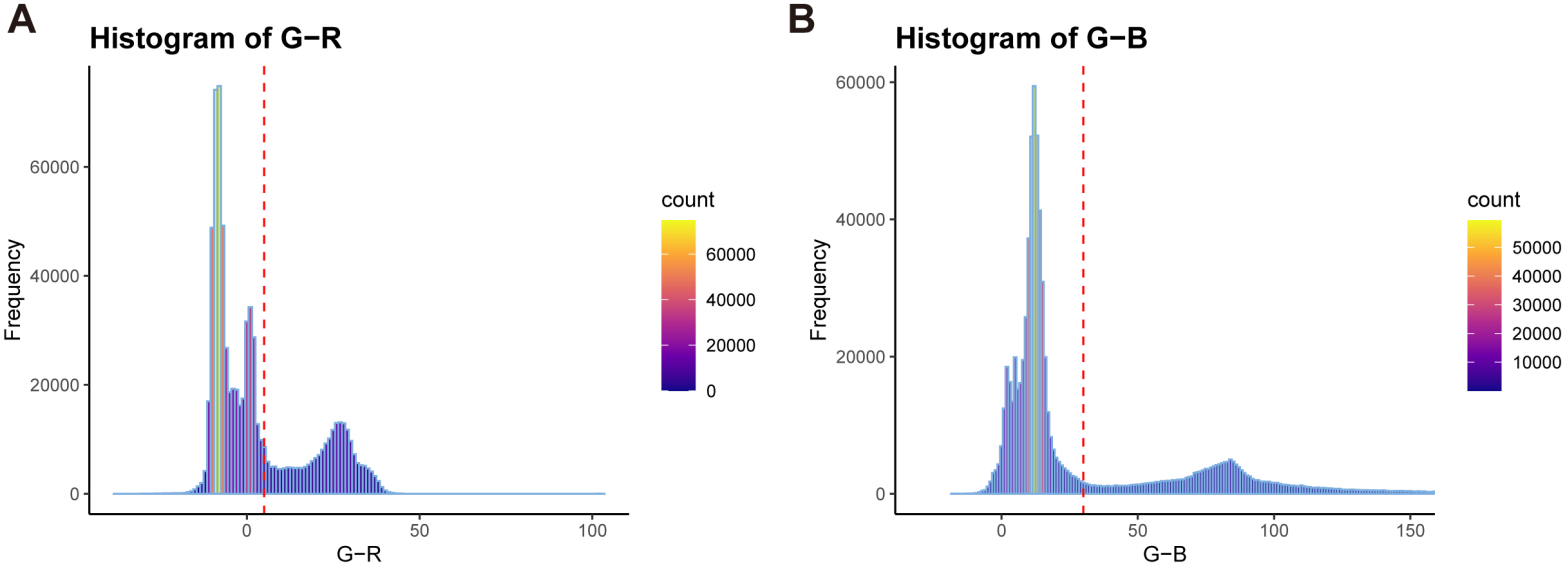
RGB color distinction frequency distribution of wheat point cloud. **(A)** G-R distribution with red dotted lines indicating G-R=5. **(B)** G-B distribution with red dotted lines indicating G-B=30.

Additionally, during the wheat image data acquisition process, numerous noise points may exist in the generated point cloud due to environmental influences and equipment accuracy. External factors such as view obscuration and obstacles can also result in unrelated discrete points and other object point clouds (**Figures 1B, C**, **Figure 1C.d**). To eliminate these irrelevant points and improve subsequent point cloud processing speed, an improved Euclidean clustering algorithm combined with centroid calculation was proposed to segment the main plant point cloud (**Figure 1C.e**). The RGB frequency distribution of the segmented point cloud was calculated (**Figure 2**), categorizing the point cloud into three clusters: soil, pot, and plant, from left to right in the R-B distribution cluster. A boundary threshold of G-R=5 and G-B=30 was set to remove most unrelated points connected to soil and pots (**Figure 1C.f**). Finally, statistical filtering was applied to remove outliers from the plant point cloud (**Figure 1C.g**). Based on above steps, a high-quality wheat point cloud was acquired, with the preprocessing algorithm workflow used in this study shown in **Figure 1C**.

### 2.3 Point cloud scaling and coordinate system correction

To accurately restore the wheat point clouds to a 1:1 scale, a scaling factor S was calculated and applied to match the real-world scale. This is done by taking the average of three measurements of the width of the marker sticker in the point cloud to obtain $W_{\text{virtual}}$ and comparing it to the real sticker width $W_{\text{real}}$ (3cm). The scaling factor S is determined using Equation 1.


(1) $$\begin{array}{c} S=\frac{W_{\text{real}}}{W_{\text{virtual}}}\; \end{array}$$

The original coordinates $\left(X_{\text{old}},Y_{\text{old}},Z_{\text{old}}\right)$ of each point were scaled according to Equation 2.


(2) $$\begin{array}{c} \begin{array}{rr} \left(X_{\text{scaled}},Y_{\text{scaled}},Z_{\text{scaled}}\right) & =\left(X_{\text{old}}\times S,Y_{\text{old}}\times S,Z_{\text{old}}\times S\right) \end{array} \end{array}$$

Next, coordinate system correction was performed by detecting the ground using the RANSAC algorithm to obtain the normal vector $n_{1}$ of the fitted plane (ground). The normal vector $n_{2}$ of the reference plane was set as (0,0,1) , representing the Z-axis direction. The rotation matrix R , was calculated to align the fitted plane with the reference plane using Equation 3.


(3) $$\begin{array}{c} R=\frac{n_{2}\times n_{1}}{∥n_{2}\times n_{1}∥} \end{array}$$

Each point in the point cloud, represented in homogeneous coordinates (x,y,z,1) , was transformed using the rotation matrix R to achieve coordinate system correction, ensuring alignment with the reference plane. The transformation process is outlined in Equation 4.


(4) $$\begin{array}{c} \begin{array}{rr} \left(X_{\text{rotated}},Y_{\text{rotated}},Z_{\text{rotated}}\right) & =\left(X_{\text{scaled}},Y_{\text{scaled}},Z_{\text{scaled}}\right)\times R \end{array} \end{array}$$

This resulted in a rotated point cloud, with the plant oriented along the Z-axis and the ground aligned with the XOY plane, ensuring accurate dimensions and coordinates for the reconstructed wheat point cloud model, providing a reliable foundation for subsequent analysis.

### 2.4 Improved Euclidean clustering algorithm combined with centroid

When processing the rotated point cloud, a considerable portion of points, such as those from the ground, lifting platform, and pots, are unrelated to the plant and constitute over 90% of the overall point cloud. Direct application of Euclidean clustering to the entire point cloud leads to extensive computational demands, prolonged processing time, and high computer performance requirements. Therefore, an improved Euclidean clustering algorithm, combined with centroid calculation, is proposed to markedly enhance segmentation efficiency. Traditional Euclidean clustering (Sun et al., 2020; Miao et al., 2023) is based on Euclidean distance metrics, where the core idea is to determine distances between points by distinguishing their proximity to neighbors, thereby clustering the spatial point cloud. To accelerate the Euclidean clustering algorithm, KD-Tree nearest neighbor search is often employed as a crucial preprocessing step. The specific steps are as follows: randomly selecting an initial point P , finding k nearest neighborhood points of P through a KD-Tree, calculating the Euclidean distance between each neighborhood point and P , and clustering points with distance smaller than the presetting threshold ( ϵ ) into the set Q . This process is iterated until the number of elements in Q stabilizes.

The improved Euclidean clustering algorithm combined with centroid calculation follows these steps:

(1) Perform Voxel Down-sampling: Apply voxel down-sampling (Sun et al., 2022) on the rotated point cloud to reduce density and computational load.
(2) Detect Ground and Calculate Centroids: Use the RANSAC algorithm to detect and mark the ground point cloud set and calculate its centroid $\left(x_{ground},y_{ground},z_{ground}\right)$ . Use color filtering to isolate the majority of the plant point cloud set and calculate its centroid $\left(x_{plant},y_{plant},z_{plant}\right)$ . (3) Determine the cut-off threshold: The cut-off threshold $Z_{threshold}$ is calculated using Equation 5.

(5) $$\begin{array}{c} Z_{threshold}=z_{ground}+\alpha \left(z_{plant}-z_{ground}\right),\;0\leq \alpha \leq 1 \end{array}$$

This threshold effectively isolates plant points from non-plant points, ensuring accurate separation.

(4) Preliminary Segmentation: Remove points below the $Z_{threshold}$ to eliminate the ground, lifting platform, and most pot points.
(5) Secondary Segmentation: Use the Euclidean clustering algorithm for secondary segmentation of the point clouds after preliminary segmentation to obtain the accurate plant point cloud set.

**Figure 3:**
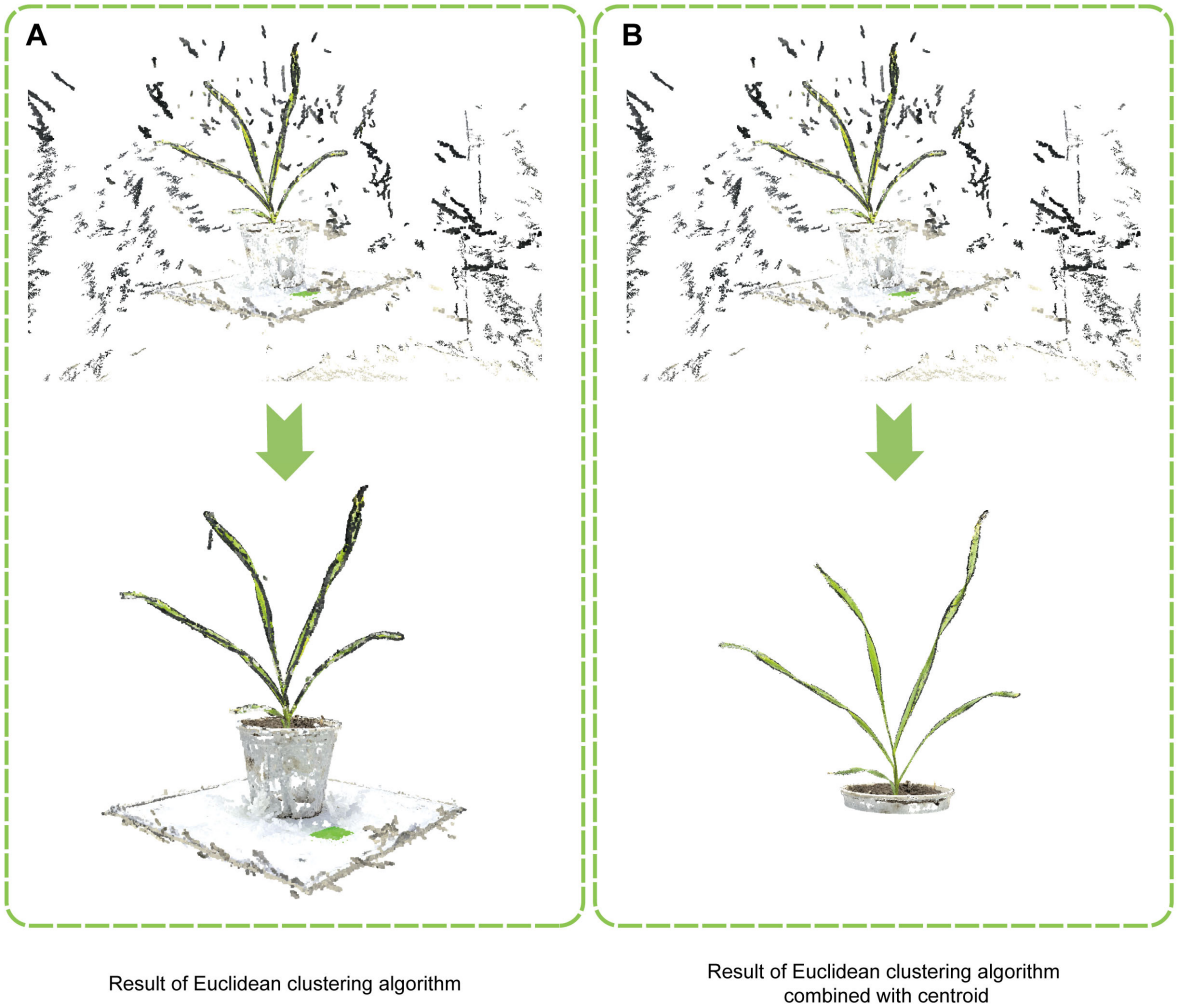
Comparison of wheat point cloud segmentation methods. **(A)** Result of the traditional Euclidean clustering algorithm. **(B)** Result of the improved Euclidean clustering algorithm combined with centroid.

Compared to the original algorithm (**Figure 3A**) , the improved Euclidean clustering algorithm incorporated centroid calculation and Euclidean clustering in a two-step segmentation process, and the implementation of adaptive determination of the cut-off threshold allowed for preliminary segmentation, reducing the volume of point cloud data (**Figure 3B**). After that, Euclidean clustering was used for fine segmentation, effectively removing irrelevant points and markedly enhancing segmentation efficiency and accuracy. These improvements enabled the efficient removal of most non-plant points, facilitating more effective Euclidean segmentation of the rotated point cloud.

### 2.5 Region growing segmentation algorithm

In this study, the region growing segmentation algorithm (Vo et al., 2015; Li et al., 2018) is employed to automatically segment wheat stems and leaves, drastically reducing the labor-intensive manual segmentation process and greatly improving the efficiency of obtaining leaf phenotypes. The region growing segmentation algorithm operates by comparing the angles between normals and curvatures of points, merging points that are sufficiently close under smoothness constraints, thereby combining similar points into regions for effective point cloud segmentation. The algorithm’s workflow is as follows:

Step 1: Calculate normals and curvatures for all points, then sort the points based on their curvature values.

Step 2: Select the point with the smallest curvature as the initial seed point. Compare the normal angles and curvature differences of neighboring points. Points meeting the predefined normal angle and curvature difference thresholds are added to the seed point’s region, while points meeting only the normal angle threshold are classified separately. Finally, all points were traversed to segment the point cloud into multiple regions with similar attributes.

### 2.6 3D phenotype extraction of wheat

Using pre-processed wheat point clouds, 3D phenotypic parameters of wheat plants were extracted, including plant height, leaf length, leaf width, crown area, plant surface area, and convex volume. Among these, plant height, leaf length, and leaf width during various growth stages were used to quantitatively assess the accuracy of the reconstructed point clouds.

**Figure 4:**
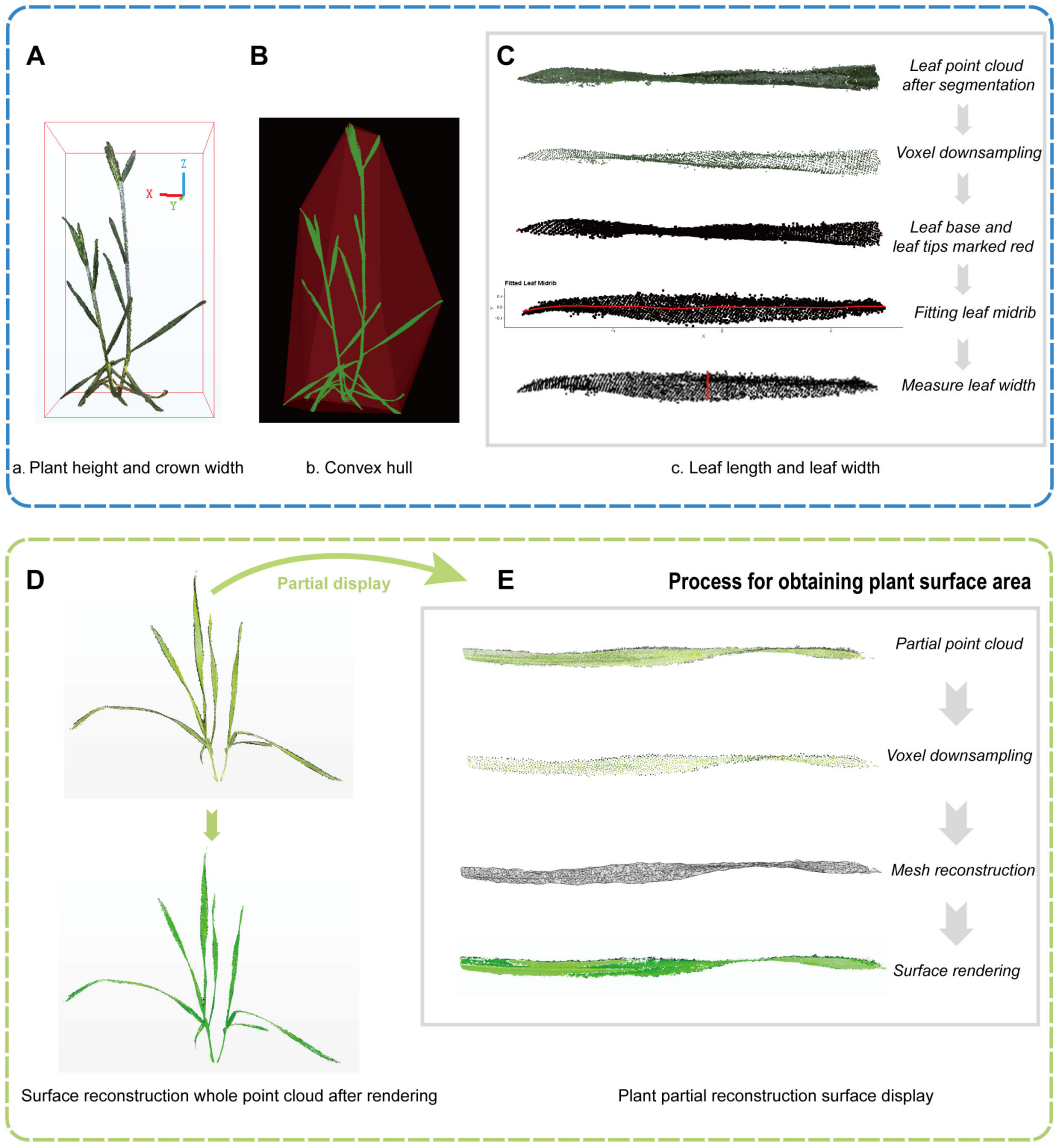
Automated extraction of 3D phenotypes in wheat. **(A)** Plant height and crown width. **(B)** Convex hull. **(C)** Leaf length and width measurement, including steps from leaf point cloud segmentation to measuring leaf width. **(D)** Surface reconstruction of the wheat plant. **(E)** Detailed view of the reconstructed surface.


Plant height serves as a crucial metric for evaluating the scaling precision of the reconstructed point clouds (Wu et al., 2022b). Crown area, plant surface area, and convex volume are essential indicators for monitoring wheat growth dynamics (Che et al., 2020; Paturkar et al., 2020). The selection of a suitable calculation method is crucial for obtaining accurate values of wheat phenotypic parameters. In this study, the following definitions and methods were used to measure key phenotypic traits:

Plant height H is defined as the vertical distance from the base of the stem at the soil interface to the highest point of the plant, represented by the height difference between the maximum and minimum Z-axis values of the plant point cloud (**Figure 4A**), given by Equation 6.

(6) $$\begin{array}{c} H=Z_{\text{max}}-Z_{\text{min}} \end{array}$$

Crown area C is defined as the product of the maximum horizontal distance of the wheat canopy projected onto the ground and its perpendicular vertical distance, which corresponds to the base area of the bounding box in **Figure 4A**. The relevant formula is provided in Equation 7.


(7) $$\begin{array}{c} C=L_{\text{max}}\times W_{\text{max}} \end{array}$$

Plant surface area refers to the total surface area of the wheat stems and leaves, calculated by reconstructing the surface area from the point cloud. The calculation process is as follows (**Figure 4E**): Voxel down-sampling of point clouds was applied (Voxel Size=0.01) to the point cloud to reduce computational load and ensure uniformity. Subsequently, the triangular mesh reconstruction was then performed using the Ball Pivoting (Bernardini et al., 1999). Finally, we calculated the sum of the areas of all triangular facets after reconstruction as the plant surface area. The left side of **Figure 4D** illustrates the originally colored point cloud and the rendered plant after surface reconstruction. Convex volume is estimated by calculating the convex hull of the plant point cloud (Wu et al., 2022b), which forms the outermost convex polygon or polyhedron enclosing the point cloud (**Figure 4B**).

Leaf length refers to the maximum surface distance from the leaf base to the leaf tip, and leaf width is the maximum surface width perpendicular to the leaf length. The automatic acquisition of leaf length is achieved by fitting the leaf midrib as follows (**Figure 4C**): (1) Apply voxel down-sampling to the segmented individual leaf point cloud to reduce computational load and ensure uniformity. (2) Conduct principal component analysis (PCA) on the sampled leaf point cloud coordinates to obtain the direction of the principal axis, then rotate the leaf point cloud so that the principal axis aligns with the X-axis, and also determining the leaf base and tip locations. (3) Use a local polynomial regression algorithm to fit the leaf midrib (Duan et al., 2016) and calculate its length. Additionally, the maximum width perpendicular to leaf length is chosen by the interactive selected point measurement method (Li et al., 2022), representing the leaf width (**Figure 4C**).

### 2.7 Statistical accuracy evaluation indicators

To evaluate the accuracy of point cloud parameter extraction and model precision, we calculated several statistical indicators including the Coefficient of Determination ( $R^{2}$ ), Root Mean Square Error ( RMSE ), and Relative Root Mean Square Error (RRMSE). These are defined in Equations 8-10.


(8) $$\begin{array}{c} R^{2}=1-\frac{\sum_{i=1}^{n}{\left(y_{i}-{\hat{y}}_{i}\right)}^{2}}{\sum_{i=1}^{n}{\left(y_{i}-\bar{y}\right)}^{2}} \end{array}$$

(9) $$\begin{array}{c} RMSE=\sqrt{\frac{\sum_{i=1}^{n}{\left(y_{i}-{\hat{y}}_{i}\right)}^{2}}{n}} \end{array}$$

(10) $$\begin{array}{c} RRMSE=\frac{\sqrt{\frac{1}{n}\sum_{i=1}^{n}{\left(y_{i}-{\hat{y}}_{i}\right)}^{2}}}{\frac{1}{n}\sum_{i=1}^{n}y_{i}} \end{array}$$

Where $y_{i}$ is the observed value (manual measurement), and ${\hat{y}}_{i}$ is the predicted value (model extracted value).

## 3 Results

### 3.1 Reconstruction and preprocessing of wheat point clouds

**Figure 5:**
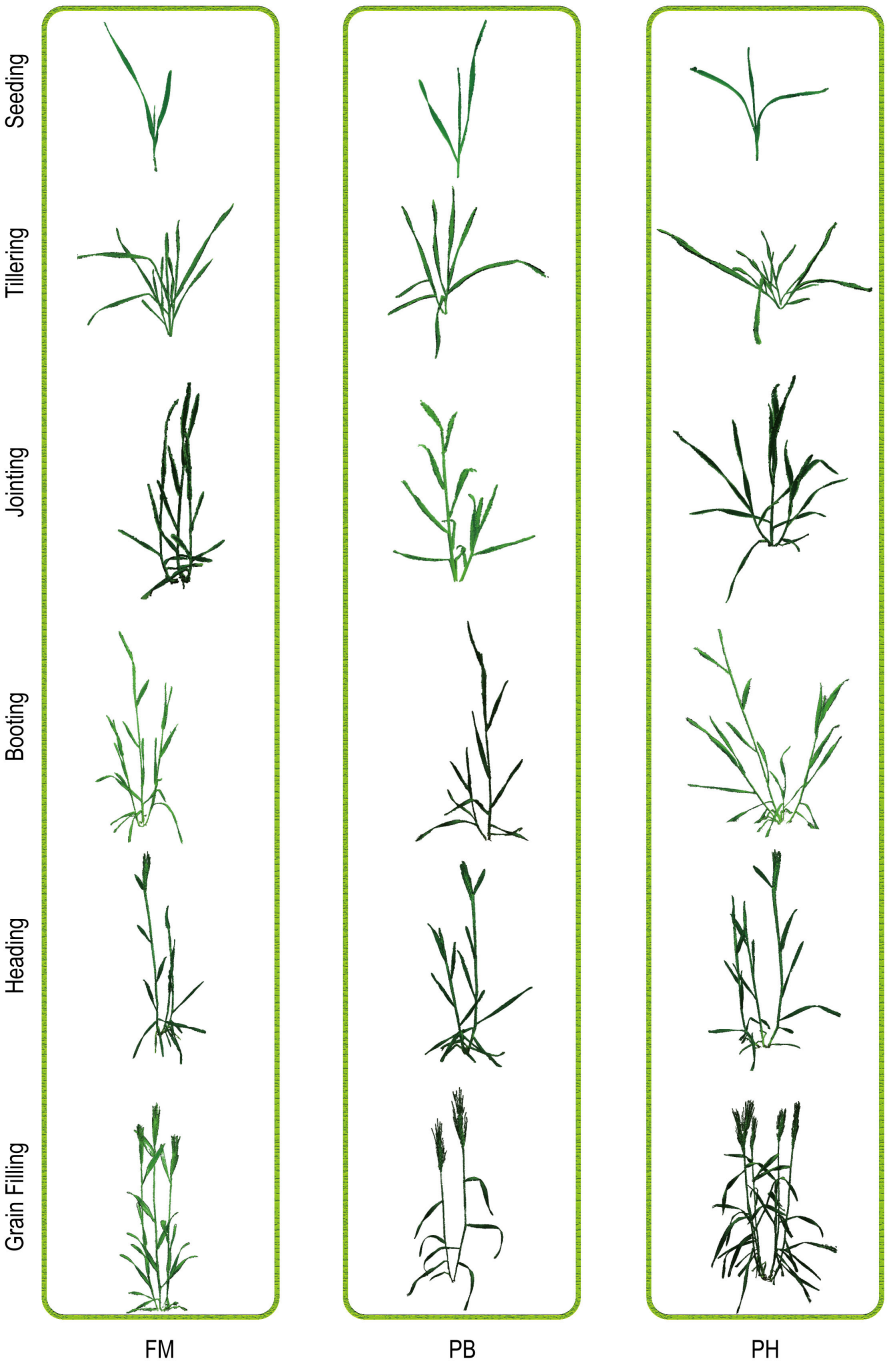
Visualization of point clouds for three wheat cultivars (FM, PH, PB) across different growth stages: seedling, tillering, jointing, booting, heading, and grain filling.

According to the preprocessing workflow of point clouds mentioned in 2.2, the point cloud data of three wheat species were meticulously processed throughout their entire growth periods, including seedling, tillering, jointing, booting, heading, and grain filling stages. This comprehensive process included scale restoration, coordinate system correction, improved Euclidean clustering segmentation, color filtering, and statistical filtering. Subsequently, the generated plant point clouds underwent triangular surface reconstruction and rendering, ensuring accurate representation of plant characteristics at each stage. The reconstruction results of the three wheat species at various stages are shown in **Figure 5**, providing a clear visualization of the 3D structures and morphological changes of the wheat plants throughout their growth periods.

### 3.2 Region growing segmentation results

**Figure 6:**
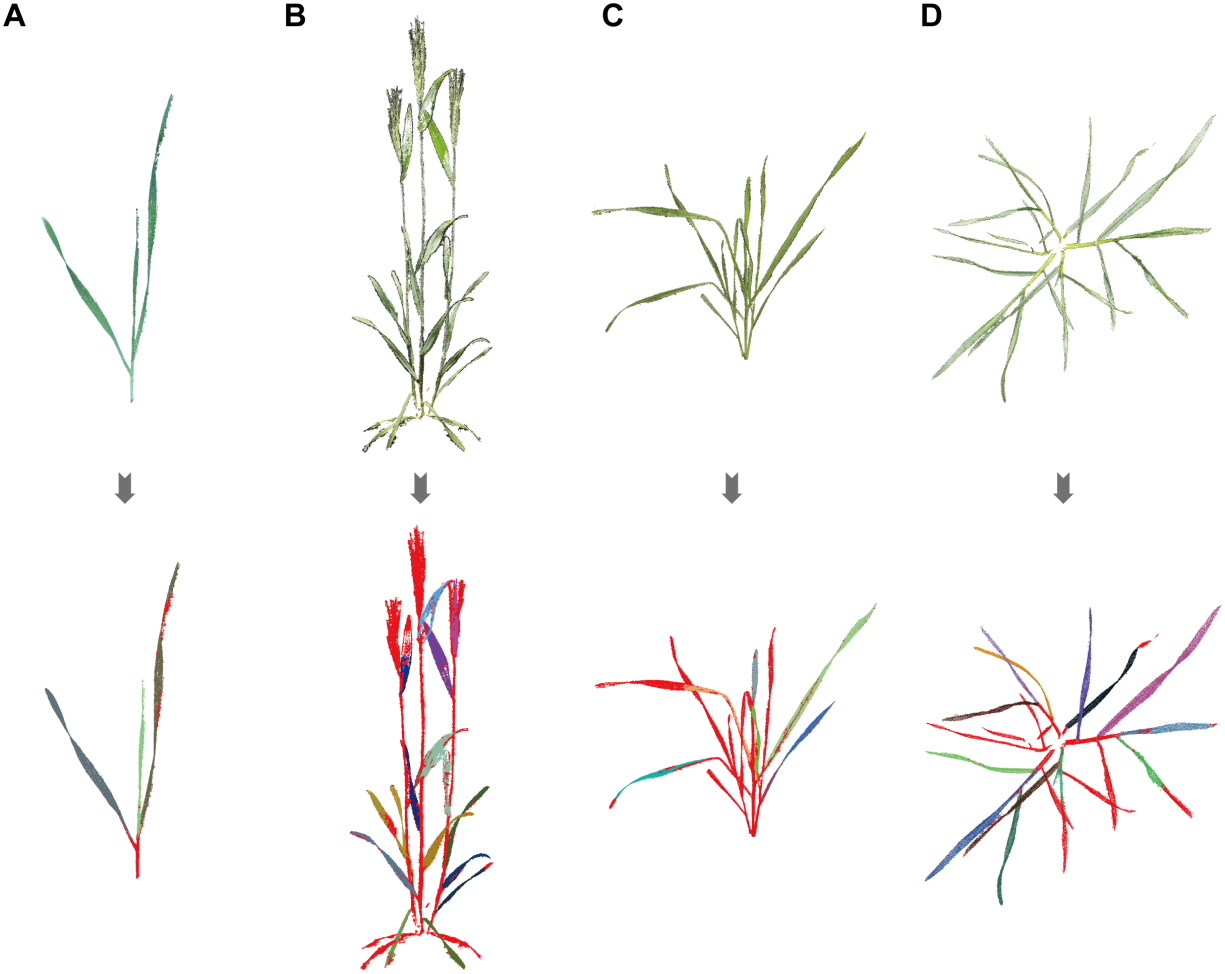
Region growing segmentation results of wheat point clouds: **(A)** Seedling, **(B)** Grain filling, **(C)** Tillering, **(D)** Jointing. Leaves are marked with different colors, and red point clouds represent unclassified points.

The region growing segmentation algorithm performed well in segmenting wheat leaves with simple and non-overlapping structures. Specifically, during the seedling stage, the algorithm accurately separated each leaf due to the small number of leaves and simple structure, as shown in **Figure 6A**, consistent with the findings of Duan et al (Duan et al., 2016; Li et al., 2022). Even during the heading and grain filling stages, despite an increase in leaf number, the algorithm remained effective due to the pronounced leaf extension (**Figure 6B**). However, during the tillering and jointing stages (**Figures 6C, D**), segmentation performance was compromised by high leaf density, severe overlapping, and leaf curling. The algorithm could not fully segment all leaves, achieving only partial segmentation of complete leaves.

Manual segmentation was employed to supplement these unsegmented parts, resulting in more complete and accurate segmentation outcomes. Although manual intervention was required, the overall efficiency of wheat leaf segmentation was considerably improved. In summary, the region growing segmentation algorithm exhibits limitations when dealing with complex overlapping leaf structures, particularly during specific growth stages. Nonetheless, combining this algorithm with manual segmentation can remarkably enhance segmentation efficiency and accuracy, laying a solid foundation for subsequent leaf phenotypic extraction.

### 3.3 Accuracy evaluation of 3D wheat point clouds

**Figure 7:**
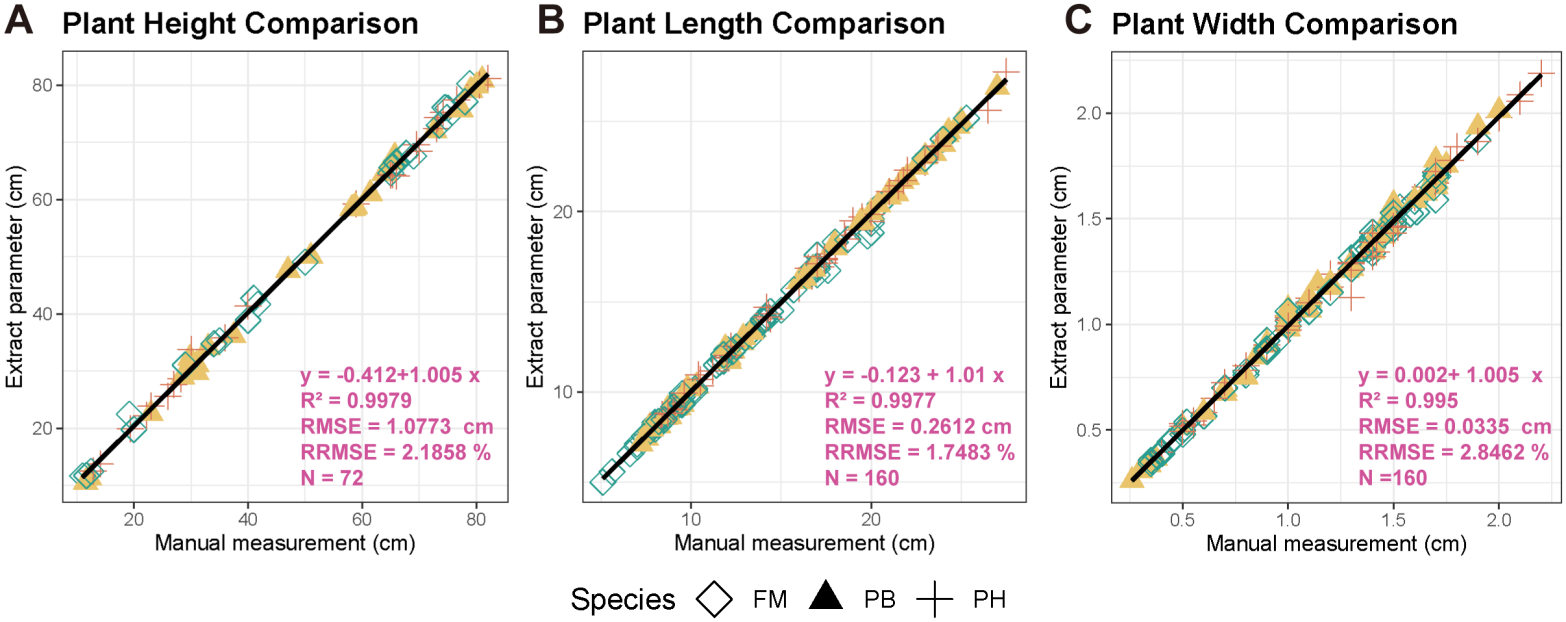
Accuracy evaluation of extracted wheat point cloud parameters. **(A)** Plant height comparison. **(B)** Plant length comparison. **(C)** Plant width comparison.

Using the phenotypic extraction methods described in section 2.6, we automatically extracted and compared 72 sets of plant height, 160 sets of leaf length, and 160 sets of leaf width with manually measured values (**Figure 7**) to quantitatively assess the accuracy of point cloud parameter extraction and model precision. The results demonstrated a significant linear relationship between the automatically extracted plant height, leaf length, and leaf width from the wheat point clouds and the manual measurements. The coefficients of determination ( $R^{2}$ ) were 0.9979, 0.9977, and 0.995, respectively; the root mean square errors ( RMSE ) were 1.0773 cm, 0.2612 cm, and 0.0335 cm, respectively; and the relative root mean square errors ( RRMSE ) were 2.1858%, 1.7483%, and 2.8462%, respectively.

By comparing point cloud measurements with manual measurements, the proposed algorithm demonstrated high accuracy in multi-view imaging reconstruction and phenotypic extraction of wheat. This also validated, to a certain extent, the practicality and stability of using 3D imaging to extract crop phenotypic parameters, enabling rapid and non-destructive extraction of plant phenotypic values.

### 3.4 Evaluation of wheat growth dynamics and variance analysis

**Figure 8:**
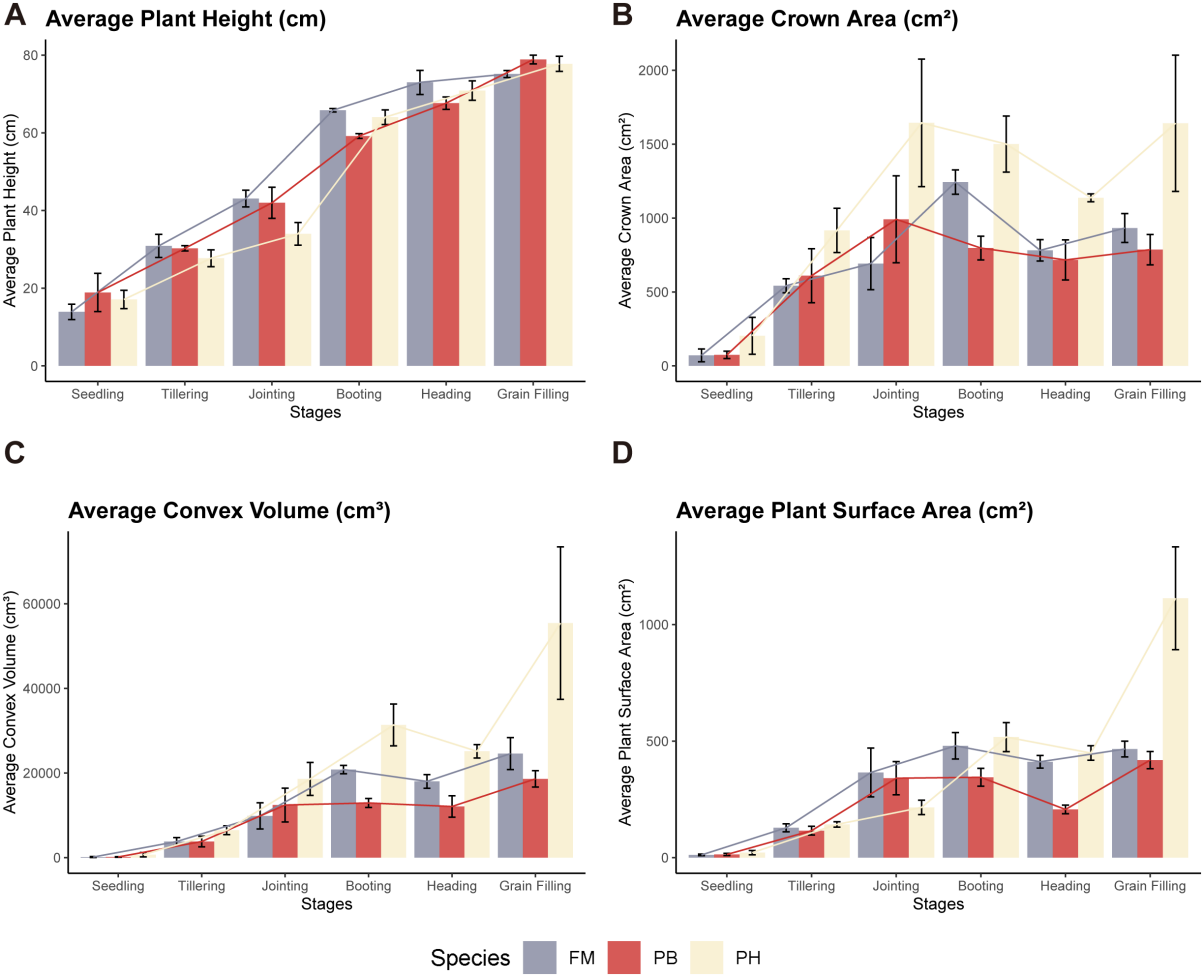
Dynamic growth changes in wheat cultivars. **(A)** Average plant height, **(B)** Average crown area, **(C)** Average convex volume, **(D)** Average plant surface area across different growth stages.

**Figure 9:**
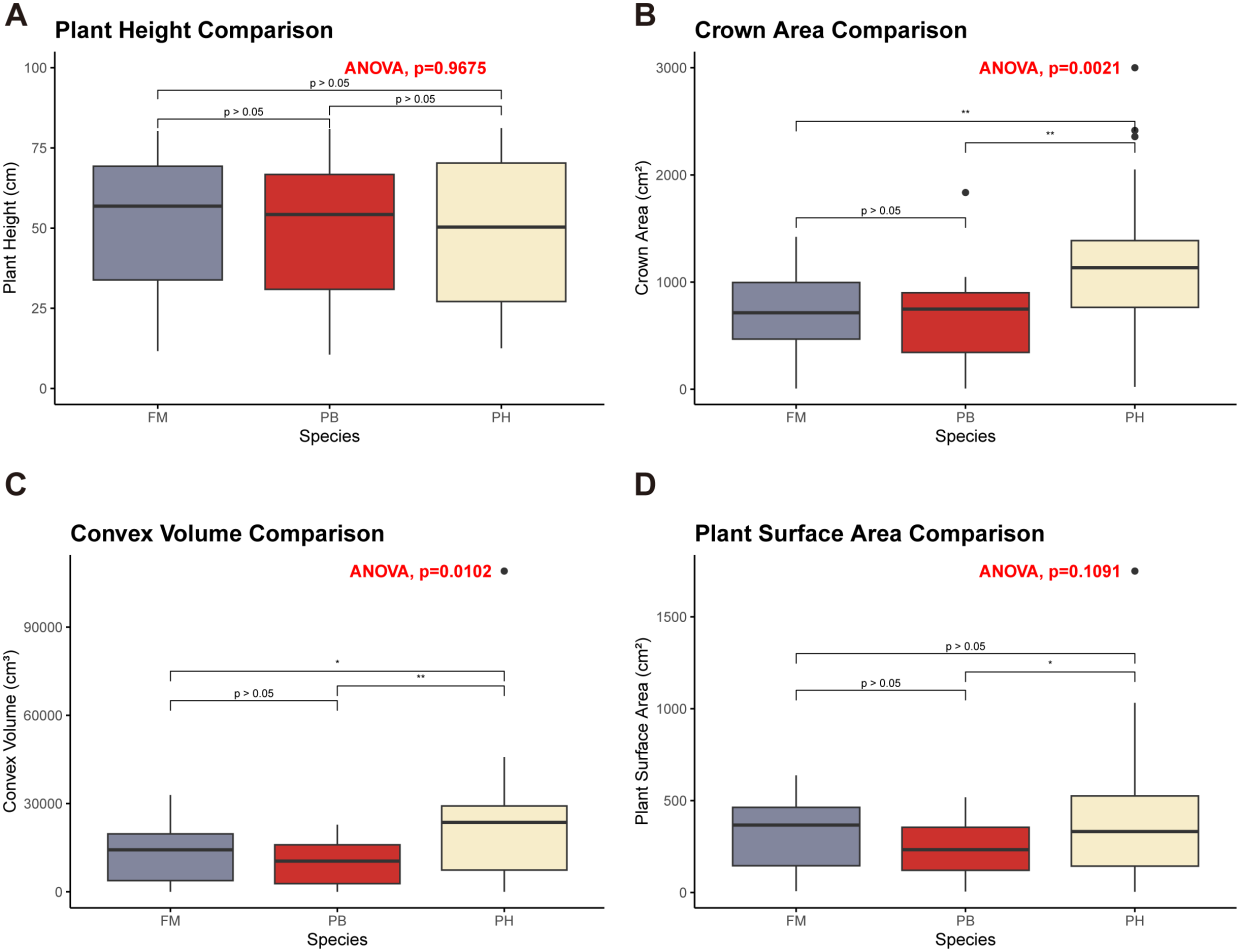
ANOVA results comparing different wheat cultivars. **(A)** Plant height comparison. **(B)** Crown area comparison. **(C)** Convex volume comparison. **(D)** Plant surface area comparison. Asterisks indicate levels of statistical significance: * p ≤ 0.05, ** p ≤ 0.01.

The average plant height, crown area, convex volume, and plant surface area of different wheat cultivars at various growth stages were meticulously extracted and calculated to reflect the growth dynamics of each variety (**Figure 8**). Meanwhile, a quantitative analysis of differences among wheat species in term of plant height, canopy area, convex hull area and plant surface area was carried out through one-way analysis of variance (ANOVA) (**Figure 9**).

Regarding plant height, there were no significant differences among the three wheat cultivars across these growth stages (**Figure 9A**, p>0.05 ). Plant height increased progressively throughout the growth cycle, with the most notable growth occurring from the jointing stage to the booting stage (**Figure 8A**). In contrast, growth from the heading stage to the grain filling stage was less pronounced.

For crown area, significant differences were observed between PH and both FM and PB ( p≤0.01 ), with PH demonstrating a larger crown area, while FM and PB showed no significant differences (**Figure 9B**, p>0.05 ). The crown area followed a trend of initially increasing, then decreasing, and subsequently increasing again throughout the growth cycle (**Figure 8B**). The turning points for PH and PB were at the jointing and heading stages, with the maximum crown area reached at the jointing stage. Conversely, FM’s turning points were at the booting and heading stages, with the peak at the booting stage.

When examining convex volume, significant differences were noted ( p≤0.05 ), with PH having a larger convex volume compared to both FM and PB, while FM and PB showed no significant differences (**Figure 9C**, p>0.05 ). The convex volume for each wheat variety exhibited a pattern of increasing, then decreasing, and increasing once more throughout the growth cycle, with turning points at the booting and heading stages, reaching a maximum at the grain filling stage (**Figure 8C**).

In terms of plant surface area, significant differences were identified between PH and PB ( p≤0.05 ), with PH having a larger plant surface area (**Figure 9D**). No significant differences were observed among the other combinations. The plant surface area of each wheat variety showed an initial increase, followed by a decrease, and another increase throughout the growth cycle, with turning points at the booting and heading stages (**Figure 8D**). PH and PB had the largest plant surface area during the grain filling stage, whereas FM reached its largest plant surface area at both the booting and grain filling stages, with minimal difference between the two stages.

Consequently, the proposed methodology effectively captures phenotypic differences across different growth stages and wheat cultivars, enabling dynamic monitoring of wheat growth status.

## 4 Discussion

With considerations to complicated features (e.g. multiple tillers, slender leaves, and organ cross-obscuration) during wheat growth, a set of method and procedure based on multi-view geometric technology was proposed in this study to process and segment wheat point clouds and automatically extract phenotypic parameters. The accuracy of phenotypic parameters for three wheat cultivars, which exhibited distinct characteristic differences throughout the growth period, was analyzed, and their growth dynamics were compared. The results demonstrate that this methodology is highly accurate and consistent, effectively monitoring phenotypic changes across the entire life cycle of wheat and the phenotypic differences among different varieties. Those discoveries have profound implications for wheat breeding and plant architecture development as well. Additionally, the study highlights the substantial potential of multi-view imaging and 3D reconstruction techniques for monitoring wheat growth dynamics and extracting high-precision phenotypic traits. Based on these findings, a detailed discussion is provided to explore the implications, limitations, and future research directions.

### 4.1 Significance of full-cycle monitoring of wheat phenotypes based on multi-view geometry

In the field of botany, traditional methods of plant phenotyping are destructive, time-consuming, inefficient, and costly, making non-destructive, rapid, and accurate acquisition of plant phenotypes a current research focus (Xie and Yang, 2020; Yang et al., 2020). Compared to 2D images, 3D reconstruction-based phenotypic extraction can express richer and more detailed 3D information (Yang et al., 2024). With the development of 3D reconstruction technology, remarkable progress has been made in crop 3D reconstruction studies using various sensors (Gibbs et al., 2016). Among these, using RGB cameras to capture multi-view images and performing 3D reconstruction based on SFM-MVS is widely applied by virtue of its low cost, strong environmental adaptability, and the capability to generate colored point clouds. This method has been successfully applied to extract high-precision phenotypic parameters for crops such as maize (Wu et al., 2020; Li et al., 2022), cotton (Hao et al., 2024), soybean (He et al., 2023), and sugar beet (Xiao et al., 2020, 2021), and others.

Compared to other crops, wheat’s growth characteristics, including dense tillers, slender leaves, and overlapping organs, make full-cycle phenotypic monitoring more complex and challenging. Previous studies on wheat phenotypic parameter extraction have used different methods. One method involves directly extracting phenotypic parameters from 2D images, such as using Mask R-CNN (Fu et al., 2021) to extract leaf length and plant height of seedling-stage wheat, with $R^{2}$ of 0.87 and 0.98, respectively. Another method involves high-precision instruments, such as LiDAR (Sun, Li et al., 2018; Forero et al., 2022; Liao et al., 2022) and 3D digitizers (Chenxi et al., 2022; Wu et al., 2022a). Zheng et al. (Chenxi et al., 2022) manually annotated key points to obtain 3D data of wheat, extracting phenotypes at different stages with $R^{2}$ of 0.98, 0.87, and 0.75 for plant height, leaf length, and leaf width, respectively. This study focuses on a simpler, cost-effective image-based SFM reconstruction method by integrating low-cost RGB cameras into our phenotyping platform. Compared to LiDAR and digitizers, this method not only captures the plant’s morphological structure but also its color characteristics, reflecting growth conditions and ensuring crop health. Our method extracts richer and more abundant information than 2D images, achieving higher accuracy and consistency in extracting plant height, leaf length, and leaf width, with $R^{2}$ of 0.9979, 0.9977, and 0.995, respectively. Next, we focused more on the application of Structure from Motion (SFM) in wheat reconstruction. Although previous studies have made notable progress in specific stages such as the seedling stage (Duan et al., 2016) (with an $R^{2}$ of 0.98 for leaf length), the greening to booting stages (Wu et al., 2022b) ( $R^{2}$ of plant height, leaf length and leaf width: 0.9991, 0.9949 and 0.9693), and the heading stage (Gu et al., 2024) (average $R^{2}$ of plant height, crown width, total leaf area and coverage: 0.78), they have yet to comprehensively characterize the growth dynamics of wheat throughout the entire growth period.

Compared to previous studies, this study used more data spanning the entire growth cycle from seedling to grain filling. By combining various algorithms for point cloud processing and phenotypic extraction, more precise and efficient wheat phenotypic extraction was achieved, comprehensively monitoring growth changes and significant differences throughout the growth period. This is of practical importance for wheat breeding and plant architecture development.

### 4.2 Accuracy assessment and error analysis

Through quantitative accuracy evaluation (**Figure 7**), a highly consistent linear relationship was identified between the wheat plant parameters extracted from the 3D point cloud model and those obtained through manual measurements. This result not only validates the reliability and completeness of the proposed algorithm for processing wheat point clouds but also confirms the accuracy of the automatic methods for obtaining plant height, leaf length, and leaf width through relatively low RMSE and RRMSE values. Compared to previous studies on wheat phenotyping, this study demonstrates a high level of accuracy, with $R^{2}$ values for all phenotypes exceeding 0.995. However, RMSE still can be improved to some extent (RMSE of plant height, leaf length and leaf width values 1.0773cm, 0.2612cm and 0.0335cm, respectively). With comprehensive and thorough considerations to experimental process, processing flow of the algorithm and previous research results, the differences in wheat phenotypic parameters may stem from several factors: 1) measurement errors in the actual data; 2) potential reconstruction deficiencies in the leaf edge regions during the point cloud reconstruction process (Duan et al., 2016); 3) possible measurement errors during the point cloud scale recovery process; 4) the potential filtering out of some plant points during the point cloud preprocessing workflow; and 5) errors in the phenotypic extraction algorithm. Optimizing these aspects is crucial for improving accuracy and represents a key direction for future work.

Furthermore, during the experiments, various quantities and distributions of images were tested to improve the resolution and quality of the wheat 3D point cloud reconstruction. The results indicated that an excessive number of images (>200) did not enhance the reconstruction resolution and sometimes resulted in poorer outcomes; conversely, an insufficient number of images (<50) led to fewer matching points, making it impossible to reconstruct the wheat plants. Considering the different characteristics of the wheat growth cycle, we recommend capturing 60-80 images during periods with fewer tillers, simpler structure and less cross-shading, and 100-140 images during periods with more tillers, more complicated structures and more cross-shading, aiming to get better reconstruction results.

### 4.3 Improvement of Euclidean clustering segmentation

Noteworthy advancements were achieved in the improvement of Euclidean clustering segmentation in this study. During the preprocessing of point clouds, the initial point cloud was found to contain a large number of irrelevant points, such as ground, pots, and lift platforms, accounting for more than 90% of the point cloud. Directly applying the traditional Euclidean clustering algorithm (Li et al., 2022) posed challenges, including high computational demands, time consumption, and substantial computer performance requirements. Hence, an improved Euclidean clustering segmentation algorithm was proposed, which remarkably enhanced segmentation efficiency and reduced algorithm runtime by incorporating centroid calculations. This improvement facilitated faster and more efficient processing of large-scale wheat point cloud data.

### 4.4 Growth dynamics monitoring and prospects

In assessing growth dynamics, ANOVA was introduced alongside traditional crop growth dynamics characterization to demonstrate significant differences among different wheat cultivars at various growth stages (**Figures 8**, **9**). For instance, PH exhibited greater crown area, higher convex hull volume and larger plant surface area than the rest two cultivars. This analysis highlights the structural differences among wheat cultivars. Moreover, trends in plant height, crown area, convex volume, and plant surface area were observed throughout the growth cycle, closely linked to physiological changes during development. Non-destructive and high-throughput phenotypic analysis of wheat plants enables early monitoring of crop structural morphology and growth conditions, providing valuable insights for wheat breeding and variety development. Our method allows for precise, automated, and high-throughput phenotyping of key growth metrics, offering breeders accurate and detailed growth data. This method can also be combined with genomic data to explore the associations between phenotypes and genotypes, thereby enhancing the selection process for superior genotypes, accelerating breeding cycles, and improving trait selection. In the realm of precision agriculture, this method can be integrated with UAV systems and ground-based sensing platforms to extract crop phenotypes and monitor crop health and development (Varela et al., 2021). Experts can use this growth and development data to provide timely interventions and prescriptions based on specific crop needs, optimizing resource use and increasing yield (Araus et al., 2022). The ability to quantify growth dynamics and trait variation also provides valuable data for crop modeling, precision agriculture, and decision support systems. Future research could focus on integrating phenotypic analysis with plant structure-function models, coupling multiple models to construct crop digital twins (Nasirahmadi and Hensel, 2022; Purcell and Neubauer, 2023), and combining crop prescription decisions to offer more precise decision support for optimizing agricultural production.

### 4.5 Limitations and prospects

The effectiveness of region growing segmentation was suboptimal during certain growth stages of wheat. Although the introduction of region growing segmentation algorithms improved segmentation efficiency and achieved some success during seedling and heading stages, overall segmentation performance was still not very satisfying during periods with extensive tillers, cross-obscuration between organs, and greater curling angles (tillering, jointing, and booting stages). The algorithm could only partially segment complete wheat leaves; moreover, achieving satisfactory segmentation results required iterative adjustments of the point cloud segmentation thresholds, which was a time-consuming process. These findings exhibit congruity with current advancements in traditional segmentation algorithms for plants. Traditional segmentation algorithms perform well on plants with few or no tillers, such as seedling wheat (Duan et al., 2016), barley (Wahabzada et al., 2015), seedling maize (Li et al., 2022), etc. However, achieving fully automated segmentation for structurally complex crops like wheat and rice remains a major challenge. Future research will focus on addressing this issue by exploring the use of neural network models (e.g., PointNet (Qi et al., 2017a), PointNet++ (Qi et al., 2017b), PointCNN (Li et al., 2018), KPConv (Kernel Point Convolution) (Thomas et al., 2019) for point cloud segmentation. Building large-scale wheat point cloud datasets for model training and optimization will help develop more effective and targeted segmentation algorithms, aiming for fully automated segmentation and improved efficiency in wheat phenotyping segmentation.

## 5 Conclusions

Considering the complex characteristics of wheat growth (e.g., extensive tillers, slender leaves, and organ cross-obscuration), this study proposed and validated an algorithm based on multi-view geometric technology. This method effectively processes point cloud data of different wheat plants at various growth stages and automatically extracts phenotypic parameters, enabling the monitoring of dynamic growth changes throughout the entire growth period of wheat. Image sequences were captured using a constructed image acquisition platform and reconstructed into wheat point clouds using the SFM-MVS algorithm. Later, wheat point clouds were extracted using a series of point cloud preprocessing algorithms. Specifically, the improved Euclidean clustering segmentation algorithm increased segmentation efficiency, reduced point cloud processing time, and effectively segmented the main parts of the plants. By comparing the extracted wheat plant height, leaf length, and leaf width with measurement results, an average $R^{2}$ of 0.9969 was achieved, indicating that this point cloud processing and phenotypic extraction method is highly accurate and efficient, making it suitable for wheat phenotypic analysis. Although RMSE still can be improved to some extent, some optimization directions are proposed to further improve accuracy and efficiency. Furthermore, through dynamic growth monitoring and ANOVA, the study demonstrated the structural growth changes and differences among different wheat varieties at various growth stages, providing valuable references for wheat plant breeding and variety development. We have recognized that the region growing segmentation algorithm performs well when wheat leaf structures are simple and non-overlapping, but its performance diminishes during periods of extensive tillers, leaf occlusion, and greater curling angles. However, future improvement directions have been proposed to achieve fully automated segmentation and enhance the efficiency of wheat phenotypic analysis.

In summary, this study proposes an effective method for monitoring the dynamic growth and phenotypic characteristics of wheat, offering insights into the relationship between plant structure and function, and optimizing agricultural production. With continued research and technological advancements, this method is expected to play a greater role in plant science and agriculture, contributing to precise and sustainable agricultural development.

## Data Availability

The raw data supporting the conclusions of this article will be made available by the authors, without undue reservation.
